# Direct and contactless electrical control of temperature of paper and textile foldable substrates using electrospun metallic-web transparent electrodes

**DOI:** 10.1038/srep34584

**Published:** 2016-10-10

**Authors:** Cristina Busuioc, Alexandru Evanghelidis, Andrei Galatanu, Ionut Enculescu

**Affiliations:** 1 National Institute of Materials Physics, Atomistilor St. 405A, Magurele, Ilfov, Romania

## Abstract

Multiple and complex functionalities are a demand nowadays for almost all materials, including common day-to-day materials such as paper, textiles, wood, etc. In the present report, the surface temperature control of different types of materials, including paper and textiles, was demonstrated by Joule heating of metallic-web transparent electrodes both by direct current and by RF induced eddy currents. Polymeric submicronic fiber webs were prepared by electrospinning, and metal sputtering was subsequently performed to transform them into flexible transparent electrodes. These electrodes were thermally attached to different substrates, including paper, textiles and glass. Using thermochromic inks, we demonstrated a high degree of control of the substrates’ surface temperature by means of the Joule effect. Metallic fiber webs appear to be excellently suited for use as transparent electrodes for controlling the surface temperature of common materials, their highly flexible nature being a major advantage when dealing with rough, bendable substrates. This kind of result could not be achieved on bendable substrates with rough surfaces such as paper or textiles while employing classical transparent electrodes i.e. metal oxides. Moreover, contactless heating with induced currents is a premiere for transparent electrodes and opens up a score of new application fields.

The interest in alternative approaches for fabricating electric or electronic multifunctional devices embedded in common objects made of cheap materials such as paper or textiles has increased steadily during the last decade^1^,^2^,^3^,^4^,^5^,^6^,^7^. A revolution is currently occurring regarding portable devices and their functionality, and smart objects are becoming ubiquitous. Alternatives are sought for niche and future applications such as foldable communication devices, “smart” advertising posters, or intelligent labels. Attaching suitable electric contacts to common materials represents a challenge because their nature prohibits the use of classical approaches currently employed in the main stream electronics industry. Metal oxides, which are the industry norm for transparent electrodes, are brittle and therefore difficult to employ on bendable substrates. Recently, several original approaches have been proposed to solve the problem of flexible or bendable transparent electrical contacts, including nanowires^5^,^8^,^9^, nanofibers^10^,^11^,^12^, nanotroughs^13^, metal meshes^14^,^15^,^16^ and lithographically designed electrodes^17^. Further such flexible, transparent electrodes made of metal nanostructures were successfully used as electrodes for controlling the surface temperature of smooth, plastic substrates^18^.

Electrospinning is a technique that allows the fabrication of continuous, uniform, submicronic polymer fibers by applying a high-intensity electric field to a polymer solution or melt droplet^19^,^20^,^21^. The resulting thin polymer fiber mats have found numerous applications, ranging from biological tissue scaffolds to filtration devices. Recently, such metal-covered mats were employed as transparent electrodes that could be easily attached to almost any type of substrate^12^,^13^,^22^,^23^. The fiber density was controlled via the preparation step and was the parameter that further determined both the electrode’s degree of transparence and its electrical conductivity. As is the case for the classical transparent metal oxide electrodes, there is a direct relation between conductivity and optical transmission.

Thermochromism is an effect that can be easily exploited in a straightforward manner for the purpose of developing particular new applications such as cheap reflective display devices, lithographical techniques being already used to design electrodes capable of controlling heating on a local, well-defined scale and on low cost substrates^17^,^24^.

In the present report, we demonstrate the possibility of controlling the superficial temperature of a substrate by means of a thin, transparent, metallic fibers web electrode via the Joule effect. This was produced either by a direct current flowing through the transparent electrode through electrical contacts or contactless by a current induced through a variable electromagnetic field. The high degree of control was demonstrated by both direct measurements and by activating thermochromic inks’ transition, a phenomenon which could potentially be further exploited in devices such as reflective displays, smart labels and posters.

## Experimental

The nanofiber webs were electrospun from poly(methyl methacrylate) solutions (PMMA, MW = 350,000, Sigma-Aldrich) using dimethylformamide (DMF, ≥99.8%, Sigma-Aldrich) as a solvent. The concentration of the solution was 10% by mass. A typical electrospinning setup was employed, where a syringe needle was used as a spinneret. Square frames with 3 cm sides and made of 1 mm diameter copper wire were employed as collectors. For increased efficiency, four such frames were simultaneously attached to a support at a distance of 15 cm from the spinneret, which was mounted onto a sweeping head scanning a length of 20 cm parallel to the collector at a speed of 1 cm/s. A potential of 15 kV was applied to the needle, and the polymer solution was fed at a rate of 0.5 ml/h using a syringe pump. The collecting time was varied between 20 and 60 min depending on the desired web fiber density. The resulting average diameter of the resulting fibers was of about 600 nm.

After the electrospinning process, the copper frames with the polymer fiber webs were covered with a metallic layer of either gold or silver using DC or RF magnetron sputtering, respectively. The thickness of the metallic layers was estimated through SEM measurements as the difference between the coated and uncoated fibers to be approximately 200 nm.

**Figure 1:**
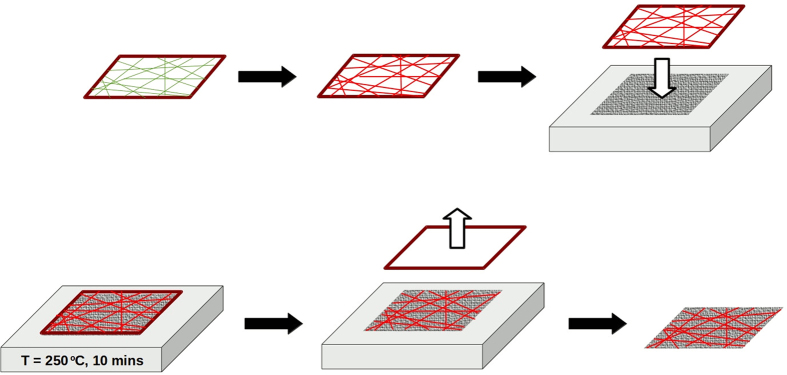
Schematic of the process for attaching the web electrodes to the substrates.

The metalized fiber meshes were subsequently transferred onto glass, textile or paper substrates (see Fig. 1 where a series of sketches of the transfer sequence is presented). Appropriately sized pieces of glass substrates were placed on a heating plate set to 250 °C, and the frames were laid directly over them and left for 10 min. In the case of the flexible substrates (textile and paper), the conductive fiber webs were attached to the substrates by heating and compression from above (with a commercial smoothing iron) to prevent sample deformation. This step led to the polymer fibers melting and acting as an adhesive of the metallic webs to the substrate. The metal electrodes are therefore well fixed and stable. Bending and long term storage do not affect samples’ electrical properties. Commercially available reversible thermochromic ink as purchased from Colour Changing was then applied to the samples with a fine paintbrush, as letters, patterns or as an arrow. The thermochromic transition temperature of the paint is 47 °C, from red to white.

Optical transmission measurements were performed for the glass-substrate-attached samples using a Perkin-Elmer Lambda 45 UV-Vis spectrophotometer equipped with an integrating sphere. The microstructure of the metalized fiber webs was analyzed with a Zeiss EVO 50 XVP scanning electron microscope. A series of electrical tests were performed to assess the conductive properties and thus the functionality of the metallic webs. The samples of gold- or silver-covered polymer fiber mats deposited onto textile or paper were therefore connected to a voltage source using aluminum foil or copper tape as intermediary contacts.

Thermal conductivity measurements were performed on both the substrates and the samples covered with metal electrodes. The measurement were performed using a LFA 457 micro flash thermal properties analyzer from Netzsch with a standard SiC 12.7 mm diameter sample holder for the perpendicular to plane measurements and a special sample holder made of stainless steel for in plane measurements on 20 mm diameter samples. In the through plane setup, a Nd-YAG laser pulse of ~3 ms is projected on the bottom surface of the sample and the temperature evolution is recorded with an InSb IR liquid nitrogen cooled detector on the opposite top surface of the sample. In the in plane setup, the laser pulse is applied on the middle of the bottom surface of the sample and the temperature excursion is recorded on the opposite site on 4 windows placed at a precise distance on a concentric ring outside the middle illuminated area. All measurements were made in 0.1 mbar Ar atmosphere with a high laser voltage (2690 V). The calibration needed for specific heat was made using a Ti 100 μm foil which was in turn calibrated with the standard sapphire sample from Netzsch. The samples have been dusted with a graphite spray before measurements in order to assure the same emissivity for samples and references materials.

Each sample was characterized with a ramp applied voltage, where the potential ranged from 0 to 2 V and the sweep rate was 1 mV/s. The low sweep rate was chosen to provide sufficient time for thermal equilibrium to be achieved through heat transfer. The approximate values at which the thermochromic transition began were also taken into account. Subsequently, “on/off” potential pulses were applied at customized values for each device, with durations of 30 s or 3 min. The “on” potential must ensure the thermochromic transition from red to white, whereas the “off” potential must provide the reverse transition. The “off” potential was chosen as non-zero to allow a direct temperature estimation based on electrical resistance measurements.

In order to test the possibility of remote heating using electromagnetic induction, Au-covered nanofiber meshes attached to paper substrates and painted with thermochromic dye were placed inside the coil of a standard induction heater. The EM field was turned on and maintained until all the dye transitioned to the white state, after which it was turned off. The current applied to the coils was varied between 100 and 200 A, while the frequency of the field was 232 kHz. The EM field was turned on and maintained until all the dye transitioned to the white state, after which it was turned off. The current applied to the coils was varied between 100 and 200 A, while the frequency of the field was 232 kHz.

## Results and Discussion

**Figure 2:**
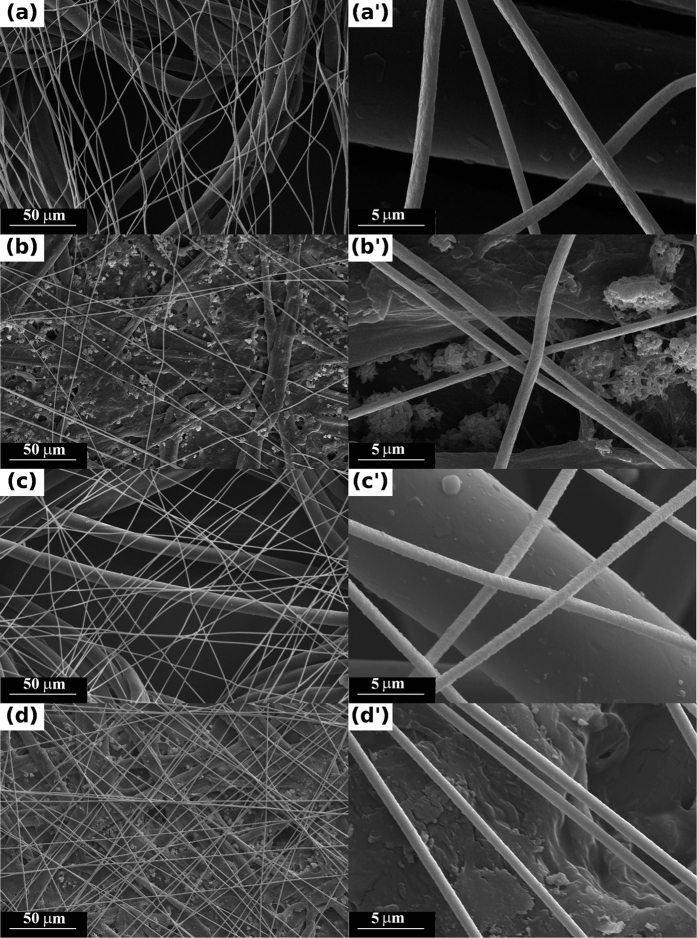
SEM images of gold-covered polymer fiber webs attached to (**a**,a’) textiles and (**b**,b’) paper and silver-covered polymer fiber webs attached to (**c**,c’) textile and (**d**,d’) paper.

The SEM images in Fig. 2 show the random fiber network uniformly covering the underlying textile or paper substrates. The fibers appear to maintain their integrity, covering uniformly large surfaces, which is extremely important when employed as electrodes. The diameter of the metalized fibers ranges between 0.9 and 1.2 μm, and the difference in diameter between the native substrate characteristic dimensions and the electrospun fibers is obvious for both the paper and the textile substrates.

**Figure 3:**
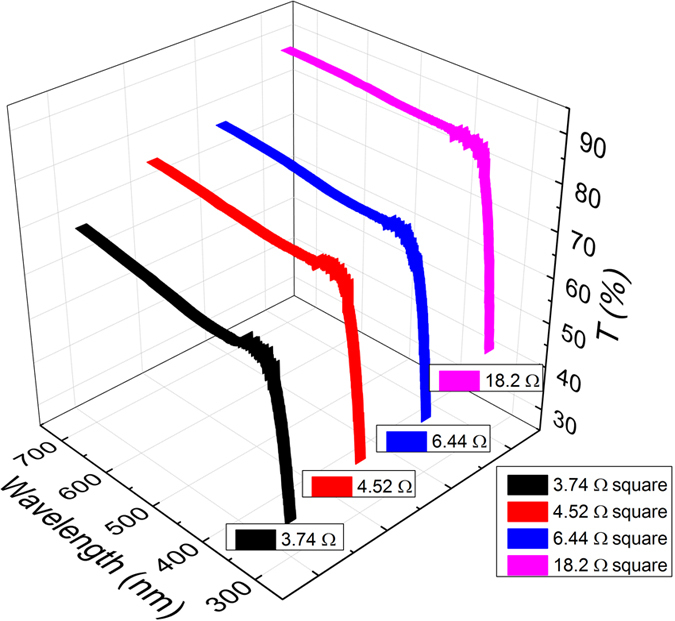
Transmission spectra of four polymer fiber webs attached to glass substrates and the correlation between transmission and resistance (inset figure).

When the process time during electrospinning was varied, layers of PMMA fibers with different densities were obtained. Given the complex, specific geometry of the samples, thickness measurements, as performed with planar electrodes, are not possible, and optical transmission measurements are better suited in the case of optoelectronic devices. The transmission spectra of a four-sample set, displayed in Fig. 3, indicate that increasing the electrospinning time leads, as expected, to a decrease in transmission in the visible range, which is a direct consequence of a thicker and denser layer of fibers. The fiber density also influences the conductivity of the metalized fiber layers, with denser networks being more conductive. The sheet resistance on a device scale was derived from the current-voltage curves of the glass-substrate-attached samples (2.5 × 2.5 cm^2^) and is correlated with their optical transparency, as evident in the inset of Fig. 3.

**Figure 4:**
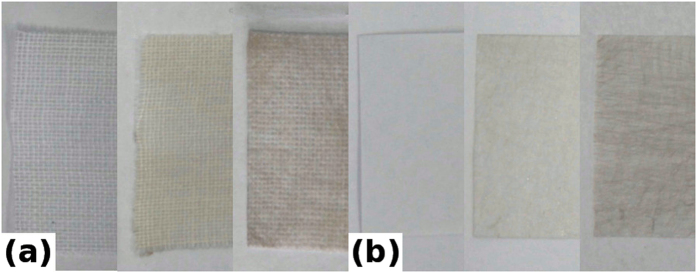
Images of substrates in pristine form and covered with metal webs: (**a**) textile (from left to right: pristine, silver covered, gold covered) (**b**) paper (from left to right: pristine, silver covered and gold covered).

With optical transmission values larger than 60%, the metal-covered fiber webs transferred onto the textile or paper substrates slightly alter their visual appearance, causing them to acquire a yellow tint for the gold covered webs and a grayish one for silver coated samples, as evidenced in Fig. 4.

**Figure 5:**
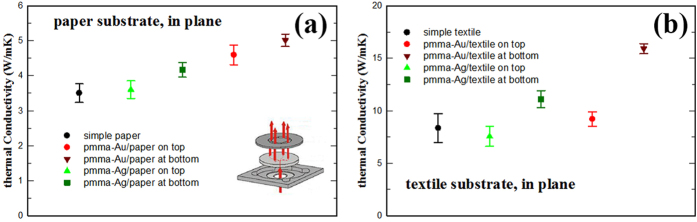
In plane thermal conductivity measurements (**a**) paper substrates and (**b**) textile substrates.

The simple paper substrate employed was measured on a transversal configuration to obtain the main parameters regarding its thermal conductivity. The density of about 0.8 g/cm^3^ and a thermal conductivity of 0.098 W/mK at room temperature of the substrate fits very well with data reported in the literature^25^. The values obtained in the transversal measuring setup have been used to further investigate the in-plane conductivity for simple and PMMA/Ag and PMMA/Au coated substrates at room temperature. The results for in plane conductivity are presented in Fig. 5 (a – paper substrate, b – textile substrate). One can observe that the values strongly differ if the metal web is the one heated first by the laser beam (at bottom) or if the metal webs are on the opposite side (on top). This can be explained by the fact that the heat is faster transferred by the PMMA/metal and start heating the reading area faster than in the case when the heat is first absorbed by paper, reach the coating and then travels through the coating toward the measuring surface window. The results with textile substrates are similar, with lower values for the bare substrate measured in transversal mode (~0.058 W/mK as compared to paper ~0.1 W/mK) but with higher values for in-plane conductivity. This might be due in part to a better adherence of the fibers on substrate. On the other side, the biggest enhancement can be observed again when the PMMA/Au coating is exposed to the laser flash, while the other samples show close values to the bare substrate. The measurements prove that a large proportion of the thermal conductivity of the composite materials in the specific arrangement is related to heat transport through the metallic web cover for both paper and textile substrates. This information is extremely important for eventual applications showing that one may use metal web electrodes for controlling the surface.

**Figure 6:**
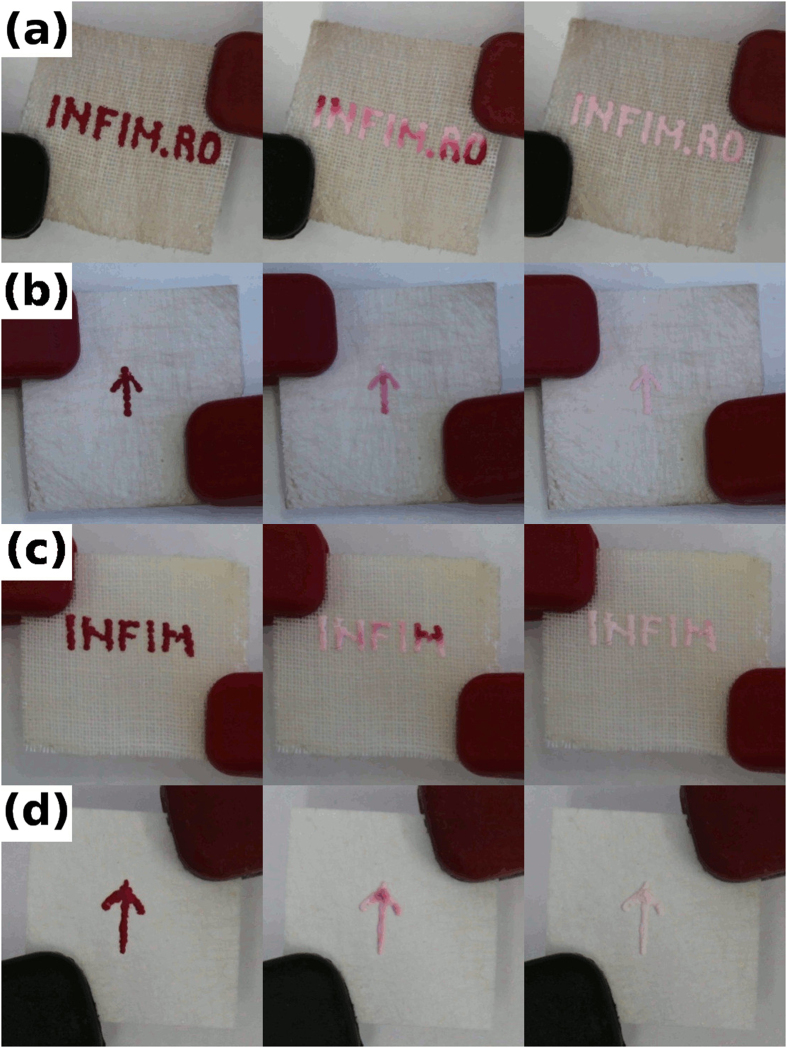
Images of gold- and silver-covered polymer fiber networks attached to different substrates; these images show the temperature transition of the thermochromic ink for (**a**) gold on fabric, (**b**) gold on paper, (**c**) silver on fabric, and (**d**) silver on paper.

Thermochromism is the ability of a substance to change its color as a function of temperature. In our study, we used a commercial reversible thermochromic ink that changes from a colored state at room temperature to a colorless state when heated and returns to the colored state upon cooling. The temperature at which the color switching occurs is called the activation temperature—in our case, 47 °C. Figure 6 presents images of gold- or silver-covered polymer fiber networks attached to textiles or paper; these images show the temperature transition of the shapes painted with thermochromic ink. An example of thermochromic transition is shown as supplementary information Video 1. The images from the first column represent the state of the thermochromic device at room temperature, with the patterns colored in red. Then, when a low voltage is applied to the metal layer covering the polymer fiber network, the temperature increases by resistive heating and the color of the thermochromic ink gradually changes, as shown in the images in the second column. Finally, when the temperature of the entire volume of thermochromic paint exceeds 47 °C, the shapes become totally white (see the images in the third column).

**Figure 7:**
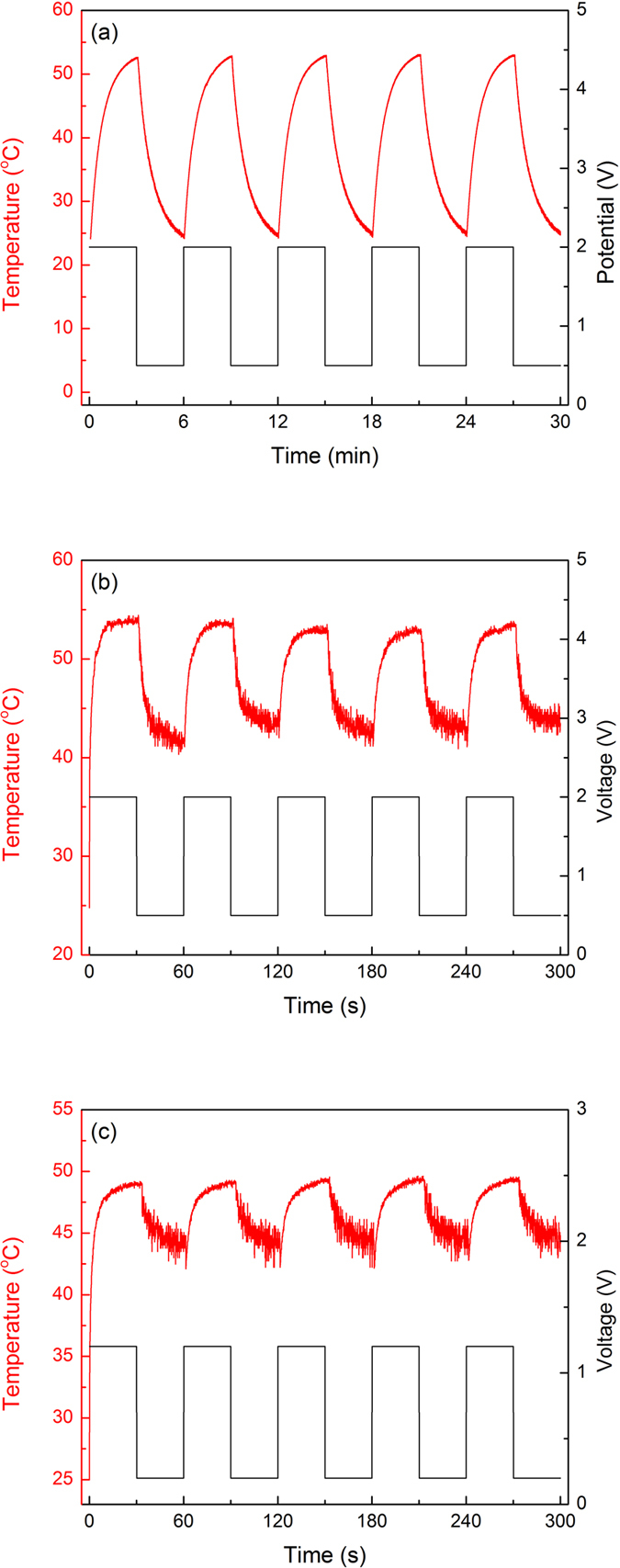
Temperature vs. time as a function of the applied voltage for (**a**) gold-covered polymer fiber networks attached to glass and for silver-covered polymer fiber networks attached to (**b**) textiles and (**c**) paper.

The temperature reached by the metalized fiber mesh was estimated using the formula *R* = *R*_*0*_ · [*1* − *α* · (*T* − *T*_*0*_)], where *R* is the resistance at temperature *T*, *R*_*0*_ is the resistance at room temperature (*T*_*0*_) and *α* is the temperature coefficient of resistance (0.0034 °C^−1^ for gold and 0.0038 °C^−1^ for silver). Figure 7a–c show the cycling for three different samples corresponding to the three substrates: gold on glass, silver on textile and silver on paper, respectively. The choice of substrate clearly influences the thermal behavior of the device because each material has a different thermal conductivity and thermal capacity as well as a different surface morphology. The smooth surface of the glass substrate provides a larger contact area with the metalized fiber network, leading to more efficient thermal transfer, which, coupled with the greater density and thermal mass of glass, promotes a slower, more gradual heating of the fibers as the substrate heats along with them.

**Figure 8:**
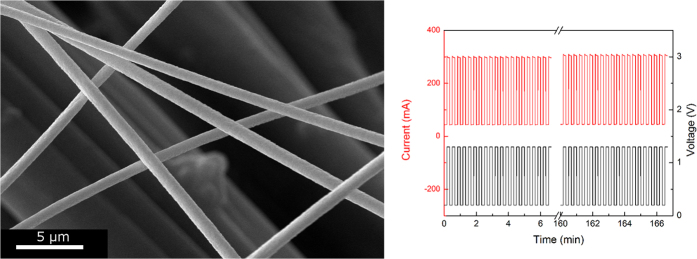
(**a**) Images of samples after 500 heating cycles (**b**) Electrical measurements over 500 cycles.

In contrast, the porous surfaces of the paper and textile substrates provide less contact area and less capacity for absorbing heat, thus favoring the rapid heating of the metalized fiber mesh, with little heat being absorbed by the substrate. As such, different substrates can be used for different practical display applications. For slow-changing, long-term information displays, a substrate with good thermal mass would be preferred because of its lower energy consumption. Fast-switching displays, however, would require better insulating substrates, which enable rapid heating of the fibers and thermochromic triggering. Notably, the heating curves are reproducible over long periods of on and off applied signals, meaning that the process is reversible and that no electrical “burning” of fibers occurs over time (see Fig. 8). In addition, the heating process is rather uniform over relatively large surfaces, which is a direct consequence of the uniformity of the metallic webs prepared by the electrospinning process.

**Figure 9:**
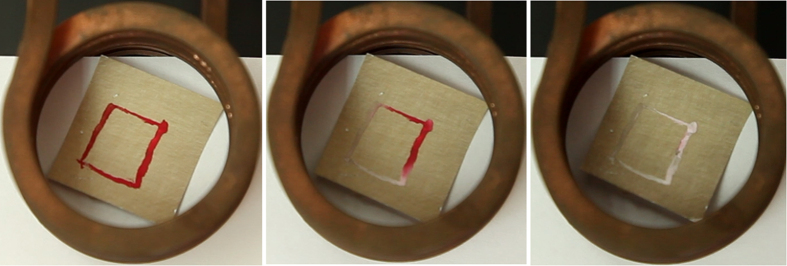
Thermochromic transition contactless induced on a gold web electrode covered paper substrate.

An induction heater setup working at 232 kHz was employed for producing a similar effect without a direct contact to the sample. During the simple on-off test conducted with the induction heater, the samples showed the same behavior as with direct contact based Joule heating – the results being visible in Fig. 9. Full transition of the dye was achieved (as shown in supplemental data, Video 2), which implies that a temperature of at least 47 °C was obtained at the surface of the fibers. As it was the case with the direct contact the sample was able to support multiple on/off cycles without any apparent damage.

## Conclusions

Precise control of the temperature of substrates such as paper and textile materials was achieved using Joule effect in metallic-fiber webs transparent electrodes. Both direct current and contactless heating can be performed. The method was employed to demonstrate thermochromic transitions in commercial inks on common substrates such as paper and fabrics, this simple technique could be adapted for developing a wide range of applications. The initial network of as-spun PMMA was covered with a layer of metal (gold or silver) and subsequently attached to different types of substrates (glass, textile or paper). Predefined configurations of thermochromic inks were then painted onto the surface of the fiber web covered substrate. By applying electrical power to the devices, we have demonstrated their functionality: a color transition from red to white when heating and the reverse transition upon cooling. The durability of the system devices was demonstrated over hundreds of “heating/cooling” cycles. Such fiber-based systems could be easily integrated into novel applications that require transparency, flexibility, low power consumption and low processing costs. Contactless heating of common materials through transparent electrodes is a premiere which opens up numerous paths for new applications.

## Additional Information

**How to cite this article**: Busuioc, C. *et al*. Direct and contactless electrical control of temperature of paper and textile foldable substrates using electrospun metallic-web transparent electrodes. *Sci. Rep.* **6**, 34584; doi: 10.1038/srep34584 (2016).

## Supplementary Material

Supplementary Information

Supplementary Video S1

Supplementary Video S2
