# Supplementary material for: Direct and contactless electrical control of temperature of paper and textile foldable substrates using electrospun metallic-web transparent electrodes
Source: Sci Rep. 2016 Oct 10;6:34584. doi: 10.1038/srep34584 (PMC5056389; doi:10.1038/srep34584)
Supplement: Supplementary Information [file srep34584-s1.pdf]

Direct and contactless electrical control of temperature of paper and textile foldable substrates using electrospun metallic-web transparent electrodes,

Cristina Busuioc, Alexandru Evanghelidis, Andrei Galatanu, Ionut Enculescu

Video Legends – (Direct and contactless electrical control of temperature of paper and textile foldable substrates using electrospun metallic-web transparent electrodes, authors Cristina Busuioc, Alexandru Evanghelidis, Andrei Galatanu, Ionut Enculescu)

Video 1. Thermochromic transition induced by surface heating by means of Joule effect in metallic webs.

Video 2. Thermochromic transition induced by surface heating by means of contacless (RF induced) Joule effect in metallic webs.
